# Analysis of the high-quality development path of China’s tea export

**DOI:** 10.1371/journal.pone.0311629

**Published:** 2024-11-12

**Authors:** Kun Qin, Lexin Zhou

**Affiliations:** 1 College of Management, Guizhou University, Guiyang, Guizhou, China; 2 College of Tea Science, Guizhou University, Guiyang, Guizhou, China; Shandong University of Technology, CHINA

## Abstract

As globalization and economic development accelerate, Chinese tea has emerged as an essential export commodity in the international market. China boasts abundant tea resources, which have significantly promoted economic growth through tea exports. This study analyses the relevant literature and the prevailing state of China’s tea export trade. Using statistical data on tea exports and empirical research methodologies, this investigation examines the key determinants influencing the high-quality development of China’s tea export sector. The research integrates relevant frontier theories from management science and engineering to propose tailored recommendations. The study reveals that while the industry has demonstrated robust economic growth, it is confronted with challenges such as the need for enhanced brand visibility and adherence to international quality benchmarks. The research findings highlight the positive influence of the "Belt and Road" initiative on the tea export trade, which advocates for strategic international collaboration. This study concludes with policy recommendations that underscore the importance of bolstering the international dissemination of Chinese tea culture, standardizing export practices, and fostering global cooperation to augment the industry’s high-quality development trajectory. The findings aim to enhance the export capabilities of Chinese tea and foster the robust growth of the tea export industry.

## 1. Introduction

The year 2023 marked a pivotal moment for the tea industry, with China’s tea market expected to surpass a valuation of one trillion yuan. The per capita tea consumption in China is projected to reach 3.5 pounds, indicating a significant increase in tea intake among the populace. Additionally, the number of tea consumers in China was set to exceed the 500 million mark. In this context, the tea industry was poised to assume a more substantial role, particularly in reinforcing the eradication of poverty and in catalyzing the revitalization of rural areas. This development would have a profound impact on more than 30 million tea farmers in China, underscoring the sector’s vital contribution to their economic well-being and broader rural economy.

With the country’s rapid economic development and increasing improvement in people’s living standards, people’s pursuit of green, healthy and convenient high-quality food consumption is also increasing [1–3]. The change in the pace of life has also promoted changes in the beverage industry, resulting in beverages becoming part of people’s lives. Among them, tea beverages are also accepted by many consumers because of their health and safety, and they are also developing rapidly in the international market. Tea leaves, the raw material of tea beverages, are also important products in the international trade market.

Tea is a national beverage, and tea planting and tea use have a long history. China is the world’s largest tea producer, consumer and foreign trade country, and tea is also one of the important elements of China’s agricultural exports [4, 5]. The country is divided into four tea regions: South China, Southwest China, Jiangnan and Jiangbei. The comprehensive tea industry output values of the provinces of interest are as follows: Fujian Province 140 billion, Yunnan Province 107.1 billion, Hunan Province 101.2 billion, Sichuan Province 100 billion, Hubei Province 71.5 billion, Anhui Province 61.5 billion, and Guizhou Province 57 billion. Therefore, fully understanding and taking advantage of the development of the tea industry and improving the level of tea exports are important. Moreover, it could seize development opportunities and effectively respond to international competition. Moreover, strong measures could be taken to stabilize the international status of China’s tea trade and better promote tea exports.

With respect to the development of the tea export industry, domestic and foreign scholars have conducted much research. The factors contributing to the expansion of Japanese green tea exports to the USA are discussed on the basis of the econometric analysis principle [6–12]. First, the demand for Japanese green tea is different from that in China and the rest of the world. The absolute value of the price and expenditure of Japanese green tea is less elastic in demand than that of Chinese green tea. Second, Japanese green tea was more popular among Americans during certain periods. The third factor is the low expenditure elasticity of Japanese green tea production. PAL et al. reported that tea exports in India have autoregressive effects and that production and export price realizations have asymmetric relationships with Indian tea exports in different quartiles [13]. Through tariff reduction and pesticide policy cooperation, the impacts of the TPP and RCEP on Vietnam’s tea exports are illustrated, and the impacts of trade barriers in tea products of member countries in regional trade agreements and excessive agricultural residues of tea products are analysed [14]. Ning et al. demonstrated the adverse impact of new EU food safety standards on Chinese tea exports. Focusing on the environment and trade sustainability, some scholars have combined economic indicators and environmental indicators to analyse the tea industry systematically [15–18]. The results revealed that the tea industry has good economic benefits and environmental protection. The least squares method, concentration index model and SWOT model were used to compare the development of the tea export trade at home and abroad in recent years [19–21]. The standardization of tea products is an important factor affecting tea exports, and increasing pressure from competition in developing countries has resulted. The goal of the development of tea products in China is high-quality, organic, characteristic and product innovation. On the one hand, the establishment of a vast area of organic tea gardens and pollution-free tea gardens should be ensured, and management should be strengthened to achieve "controllable, traceable and guaranteed" to ensure high-quality tea exports. Sun et al. discovered a robust positive link between institutional quality and energy efficiency, including the beneficial spillover effects from neighboring countries’ institutional strengths [22, 23]. Liu et al. utilized grounded theory and csQCA to dissect China’s green finance policy, identifying key governance elements and optimization strategies for sustainable development [24]. Suroso et al. used the autoregressive distributive lag from 1991–2020 to evaluate the effect of China’s tea exports on economic growth [25]. A panel data (2010–2019) methodology was adopted using 10 Doing business indicators from the World Bank and total early-stage entrepreneurial activity from the Global Entrepreneurship Monitor by Nave and Ferreira [26]. Research on the impact of policy and institutional quality on green finance and energy efficiency has important implications for the adjustment and upgrading of the tea industry. Existing studies have analysed various factors that restrict the high-quality exports of industrial industries but lack a systematic summary of the problem and propose corresponding solutions.

Therefore, in this paper, complex network analysis methods, behavioral economics, and diamond-based theory were used to analyse the export status of the tea industry. We also analyse the factors related to tea exports from different aspects, such as indicators such as the display comparative advantage index, a review of tea product exports, empirical case studies, and targeted suggestions. Through comprehensive analysis and research, this paper provides theoretical support and practical guidance for the high-quality development of China’s tea export industry. The article systematically progresses from the introduction, through a range of research methodologies, to the presentation of results and data analysis. It then delves into discussions on strategies for high-quality development before concluding with a summary of findings and their implications.

## 2. Research methodology

This scholarly work presents a holistic investigation leveraging a difference-in-differences (DID) model, sophisticated network analytical techniques, neuroeconomic inquiries into prosocial preferences, and the diamond framework for analysis.

### 2.1 Analysis of the DID model in the context of Belt and Road

Fan et al. opted for the DID model as their analytical approach [18]. They utilized the volume of Chinese tea exports as the key explanatory variable and incorporated two additional dummies: a strategic dummy to indicate Belt and Road Initiative (BRI) membership, coded as 1 for member countries and 0 for nonmembers, and a time dummy to account for temporal variations. To ensure comprehensive control for variances among nations, a suite of macroeconomic and demographic control variables was selected. These included gross domestic product (GDP), the inflation rate, the unemployment rate, the economic freedom index, and the human development index (HDI). These controls were designed to mitigate the influence of country-specific factors, thereby enhancing the robustness of the study’s findings and ensuring a more accurate estimation of the effects under investigation. The explanatory variable model for China’s foreign tea export volume was as follows::

$$\begin{array}{c} TeaTradeValue_{it}=b_{0}+b_{1}Treat_{i}+b_{2}Post_{t}+b_{3}Treat_{i}\times Post_{t}\\ +b_{4}Controls_{it}+g_{t}+e_{it} \end{array}$$ (1)

Within the analytical framework, the variable denoted as ’Tea Trade Value’ served as the independent variable, quantifying the economic magnitude of China’s tea exports to international markets. Concurrently, the term ’Treat’ was operationalized as the strategic dummy variable, which was an indicator of a country’s affiliation with the Belt and Road Initiative. where membership was binary-coded as 1, indicative of participation, and 0 signifies nonparticipation. "Yes" takes a value of 1, and "No" takes 0. "Post refers to the time dummy variable, and Treat×Post refers to the dummy variable measuring the effect of the group policy. Where i denotes the regional dimension and t denotes the time dimension. For example, the volume of Chinese tea exported to country i in year t was denoted as the tea trade value. The results indicated that the "Belt and Road Initiative" has had a significant positive effect on China’s tea export trade.

### 2.2 Analysis of complex network analysis methods

Originating within the mathematical domain, complex network analysis has undergone significant advancements, largely attributed to the small-world experiments and the introduction of scale-free network models [17]. As network topology has evolved alongside advancements in data visualization techniques, complex network analysis has been extensively applied to investigate various types of flows across different spatial scales, including trade, urban economic activity, transportation, and population movement. This approach involves abstracting the carriers of research interest as network nodes and representing the interactions, or "flows," between these carriers as edges connecting these nodes, thereby constructing a network framework. The establishment of network relationships is facilitated by representing these "flows" as lines connecting points, which allows for a comprehensive mapping of the intricate interconnections within the system under study.

C = {i, j} denotes the set of countries, i and j are trade relations with each other, and R = {r_ij_} denotes the set of tea trade volume between countries, so as to construct the i × j trade matrix W (Eq (2)). Let the actual trade links be m, and since not all countries produce tea trade links, i × j > m. The complex network analysis method includes indicators such as network density, node degree, node strength and intermediary centrality.


$$W_{i\times j}^{□}=\left[\begin{array}{l} r_{1,1}^{□}\cdots r_{1,j}^{□}\\ r_{i,1}^{□}\cdots r_{i,j}^{□} \end{array}\right]$$ (2)

Many tea-exporting countries are tea-producing countries, and the resource endowment and production conditions are the basis of tea export. The high level of tea processing capacity is value gain, which is also an important factor related to the status of tea trade. The level of national economic and social development and people’s tea drinking habits are also internal motivations for increasing tea imports. From the perspective of China’s tea export development, the proportion of bulk tea is large, the proportion of exports of famous tea is not high, and the status quo of "no brand" or "weak brand" affects the enhancement of tea value.

### 2.3 Neuroeconomic study of social preferences

Neuroeconomics, rooted in the field of neuroscience, has advanced the integration of emotional dimensions into the fabric of economic modelling, thereby extending the concept of bounded rationality. The advent of the digital economy, in tandem with the maturation of big data analytics and cloud computing technologies, has endowed neuroeconomics with richly empirical data, significantly accelerating its evolution. The growing acumen of merchants in deciphering consumer behavior was now palpable, transforming the consumer experience into one that is not only more convenient but also, to an extent, gratifying.

This study proposed the application of functional near-infrared spectroscopy (fNIRS), a sophisticated brain imaging modality, to quantify and map neuronal activity within the prefrontal cortex during risky decision-making episodes. The goal was to escalate the analytical depth from conventional "behavioral‒psychological" frameworks to a more nuanced "brain‒neuron" paradigm. This paradigm shift is underpinned by the recognition that the human brain operates through two distinct yet interwoven systems during decision-making: a goal-directed, reflective, and computationally governed "controlled system."

Moreover, the confluence of neuroeconomic and behavioral economic methodologies was harnessed to aggregate and scrutinize consumer preferences for a spectrum of export-oriented products. This analytical approach was designed to deepen the comprehension of consumer motivations and inclinations, particularly within the domain of tea exports. The insights gleaned from this study can be used to innovate marketing paradigms and enrich consumer engagement strategies.

### 2.4 Diamond theory

**Fig 1:**
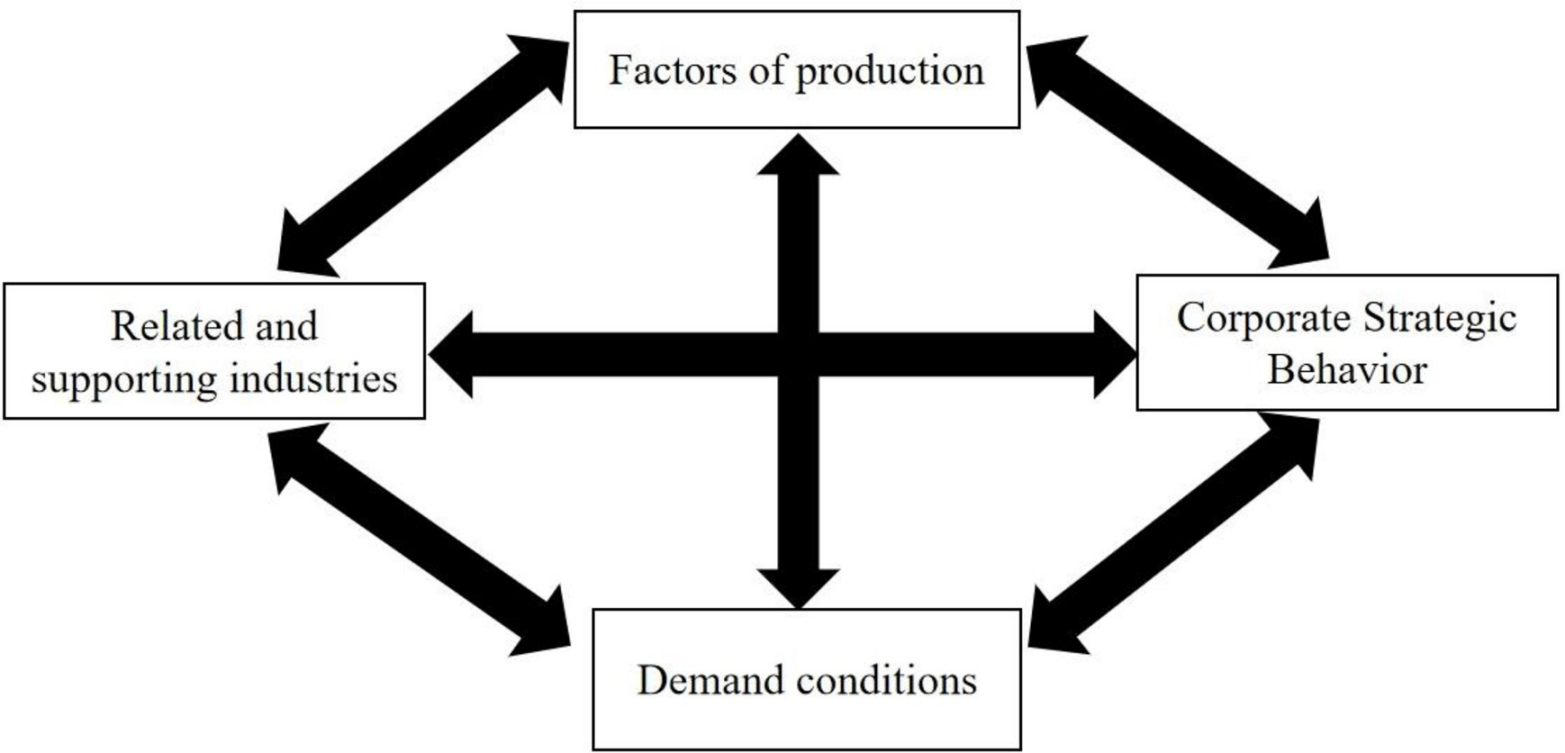
Illustration of the commonality of theories Michael Porter’s diamond theory.

To scrutinize the determinants of China’s international competitiveness in the tea sector, an in-depth analysis and study of the diamond model was employed, a theoretical framework originally conceptualized by Professor Michael Porter. The diamond model encompasses a comprehensive examination of industries ranging from primary to tertiary, focusing on specific agricultural products, including those that are traded internationally and factors pertinent to agricultural development and production.The study delves into the intrinsic mechanisms influencing the international competitiveness of China’s tea industry by examining the interplay between production factors, related and supporting industries, the strategic behaviors of firms, and the prevailing demand conditions. The analysis of these four dimensions, as posited by the diamond model, is presented as an integrative theory where the elements mutually reinforce and intersect, culminating in a diamond-shaped model that encapsulates the industry’s international competitiveness. To foster a clearer understanding and application of Michael Porter’s theory of competitive advantage, the Diamond Model theoretical framework diagram is provided as a potent visual and conceptual instrument, as shown in Fig 1.

## 3. Results and data analysis

The historical narrative of Chinese tea exports has had an enduring global impact, with China dominating the world tea market supply until the mid-19th century. Notably, during the Qing Dynasty in the 18th century, the export sales of tea began to scale up significantly, culminating in a peak in 1886. This was followed by a period of decline that lasted until the 1980s, after the liberation of New China, when the tea industry experienced a resurgence and both production and export sales flourished.

The scholarly inquiry into the export of tea in China has been multifaceted, encompassing (i) an examination of the development and trade dynamics of tea between China and the West from a Chinese perspective; (ii) studies focusing on regional markets such as China and the United States, China and Russia, and China and Britain, as well as broader research into the tea trade between China and the West; (iii) investigations into specific tea-producing regions within China, including Fujian, Hubei, and Guizhou; and (iv) comparative studies of tea-producing nations such as India and Japan in relation to Chinese tea, particularly in the late 19th century, after the established patterns of tea production and marketing were disrupted.

**Fig 2:**
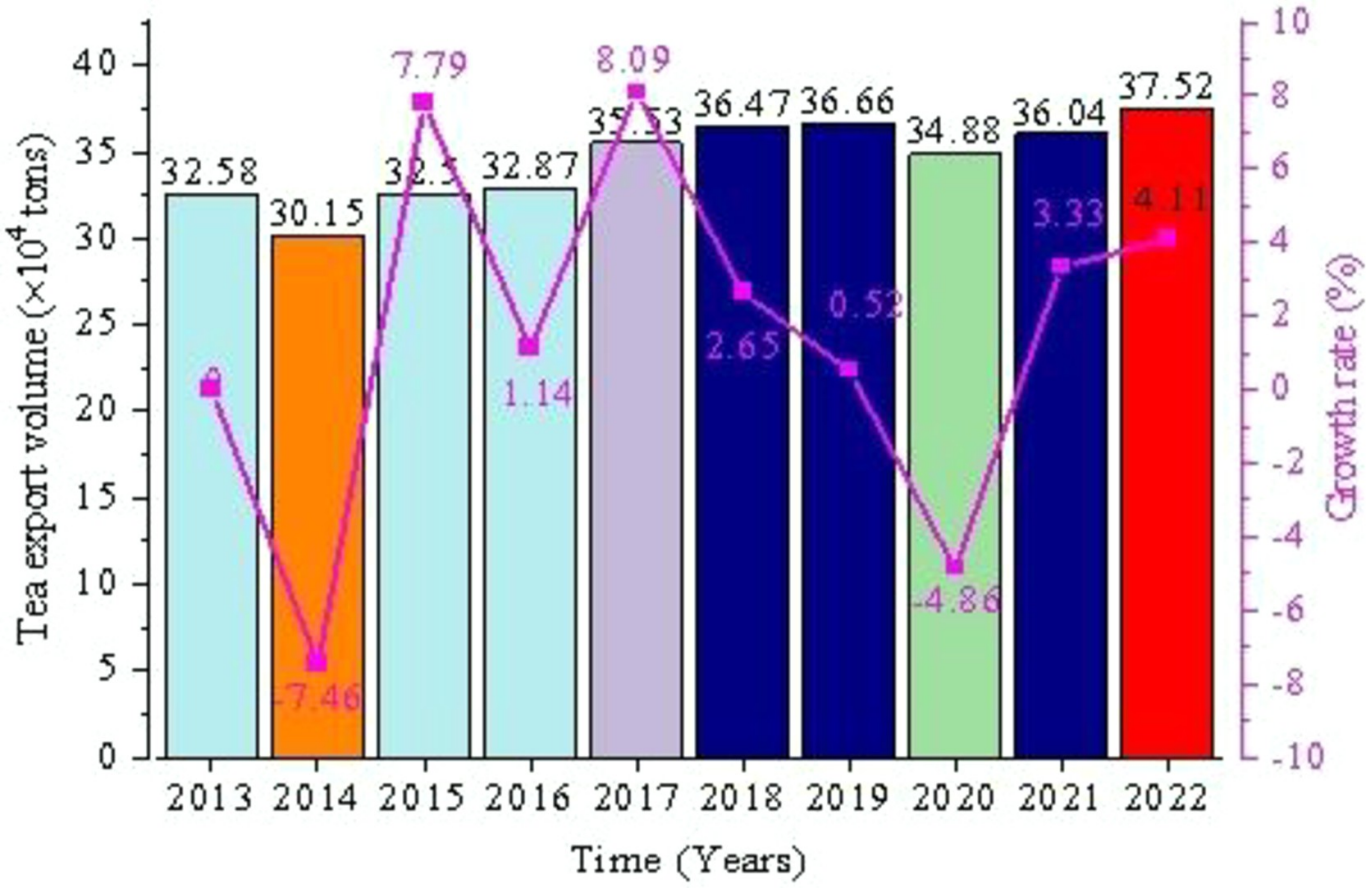
Changes in China’s tea export volume from 2013–2022.

**Fig 3:**
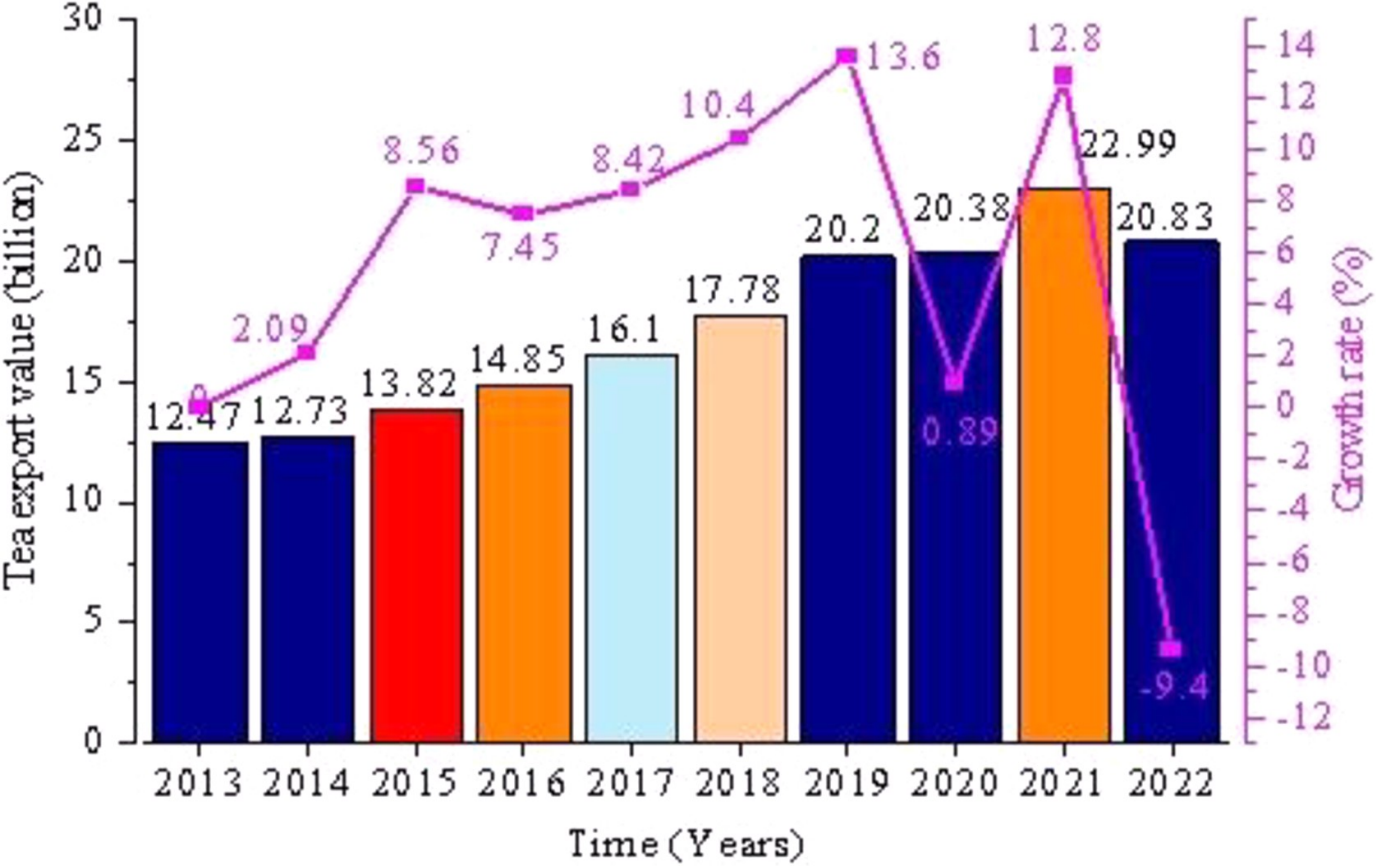
Change in China’s tea export value from 2013–2022.

From January to December 2022, China’s tea exports totaled 375,200 tons, an increase of 14800 tons or 4.11% over the whole year of 2021, as shown in Fig 2. However, the value of China’s tea exports was $2.083 billion, down $216 million from 2021 and down 9.4% year-over-year, the first negative growth in tea exports in a decade (as shown in Fig 3).

In recent years, the trajectory of China’s tea export sector has been characterized by a blend of dynamics. Despite the presence of numerous uncertainties, the export initiative has demonstrated a robust capacity for advancement, as evidenced by an overall stable and modestly increasing volume. However, the pattern of international trade in Chinese tea has not undergone significant breakthroughs. The promotion of products enriched with technological content and cultural value in the global market has been limited, resulting in exports that are often categorized as low quality and priced accordingly. Nevertheless, the Chinese tea industry possesses considerable untapped potential for improvement and growth.

An analysis of the international competitiveness index of China’s tea exports was conducted, employing pertinent indicators and market share data. This examination delves into the intrinsic correlations and projected trends, offering insights into the underlying dynamics and the path forward for China’s tea export industry on the global stage.

### 3.1 Revealed Comparative Advantage (RCA)

The RCA index is a common indicator used to measure the level of competitiveness of a country’s products or industries in the international market. It is the ratio of the share of a country or region’s exports of a certain type of product in its total exports to the share of the world’s total exports of that type of goods in the world. The larger the index is, the more competitive the country is in the international market.

The formula for the index of revealed comparative advantage is RCA=(Xat/Xa)/(Xtw/Xw). Where Xat is the export value of tea in a region, Xa is the export value of all products in a region, Xtw is the global export value of tea, and Xw is the total foreign sales of all commodities in the world. When RCA<0.8, the export competitiveness is weak; when 0.8<RCA<1.25, the export competitiveness is average; when 1.25<RCA<2.5, the export competitiveness is strong; when RCA>2.5, the export competitiveness is very strong.

By consulting the China Statistical Yearbook and the United Nations statistical database to calculate the index of the displayed comparative advantage of Chinese tea, the RCA index of tea has generally increased. However, the rate of increase is relatively flat, which indicates that Chinese tea exports have a relatively strong comparative advantage and that tea exports are competitive.

### 3.2 International Market Share (IMS)

IMS is the proportion of the total exports of a certain product of a country or region to the total exports of the world. The index is relatively simple to calculate, but can directly derive the international competitiveness results of a certain product of a country or region.

The formula for calculating the international market share is IMS = Xt/ Xw×100%, where Xt is the number of tea exports in region t and Xw is the total amount of tea exports worldwide. The larger the IMS value, the greater the export competitiveness of the tea industry in that region.

Through the use of relevant data to calculate the international market share of Chinese tea, the IMS value of tea continues to rise. The findings show that the international market share of Chinese tea in general still tends to increase annually, and the competitive advantage of tea exports is relatively obvious.

### 3.3 Trade Competition Index (TCI)

The formula of the trade competition index is TCI = (X-M) /(X+ M), where X denotes the total value of tea exports in a region and M denotes the total value of tea imports in a region. When X is greater than M, it means that the tea export of the region is greater than the import, TCI is positive and has a competitive advantage in terms of exports, TCI is large, and the tea export is competitive.

### 3.4 Data analysis

To explore the development of tea in relevant countries in recent years, data, and tea market analysis, China and the relevant tea-exporting countries’ tea exports were compared, as was the general situation of China’s high-quality tea development.

**Fig 4:**
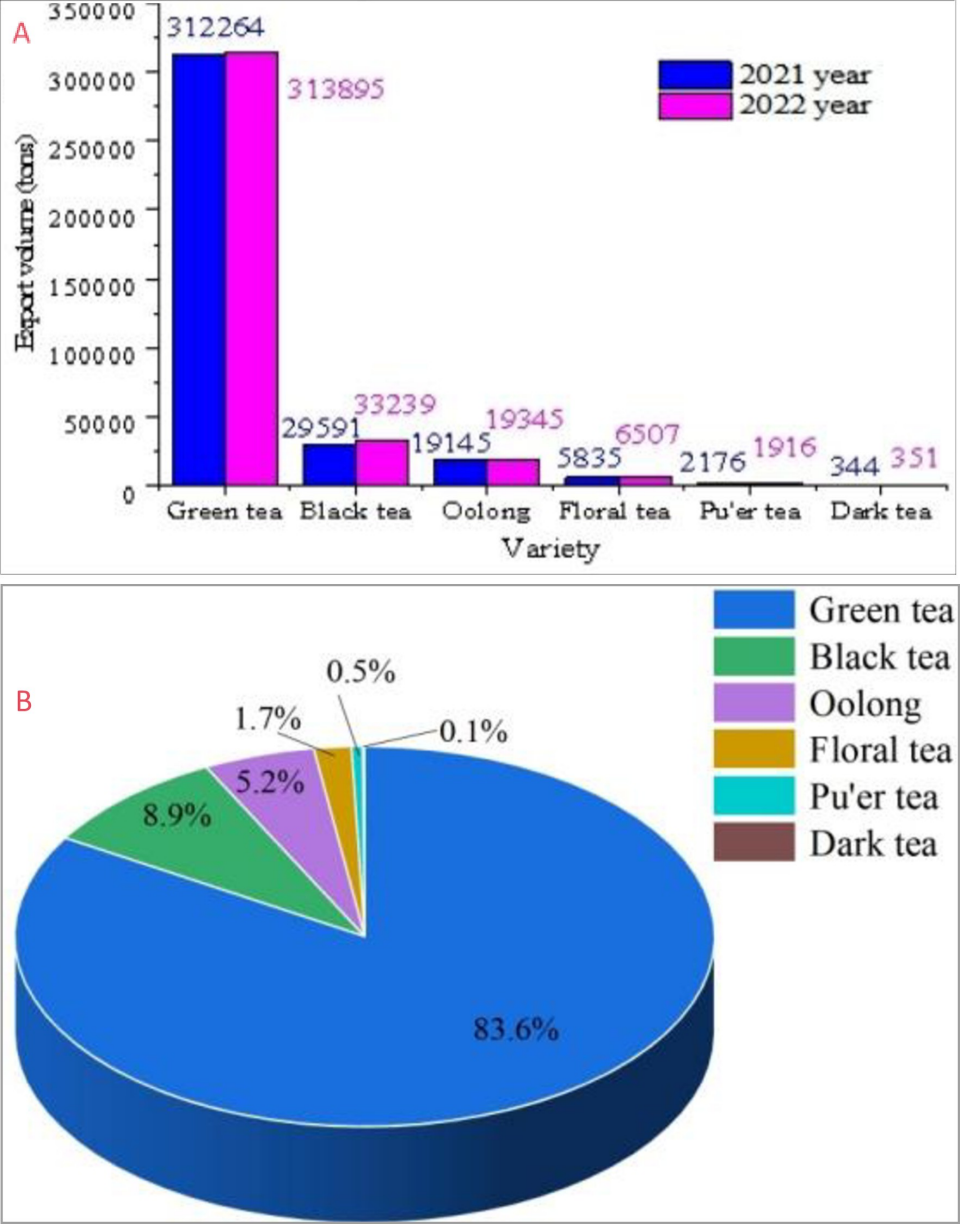
Exports by tea category. (a) Export volume comparison chart in 2021-2022(b) Pie chart of export volume in 2022.

In 2022, Chinese green tea exports were 313895 tons, accounting for 83.6% of total exports, an increase of 1631 tons, or 0.5%, as shown in Fig 4A and 4B. Black tea exports were 33200 tons, accounting for 8.9% of total exports, an increase of 3648 tons, or 12.3%; oolong tea exports were 19200 tons, accounting for 5.2%, an increase of 201 tons, an increase of 1.1%; flower tea exports of 6507 tons, accounting for 1.7% of total exports, an increase of 672 tons, an increase of 11.5%; Pu’er tea exports of 1916 tons, accounting for 0.5% of total exports, a decrease of 260 tons, a decrease of 11.9%; and black tea exports of 351 tons, accounting for 0.1% of total exports.

## 4. Discussion of the high-quality development strategy of the Chinese tea export industry

### 4.1 Management reinforcement and risk control

Gather early-warning intelligence and enhancing risk mitigation strategies: Export tea companies should vigilantly monitor specialized platforms for risk alerts to stay abreast of the latest technical trade measures. Timely acquisition of foreign inspection, quarantine technology, and risk warning information is crucial for preemptive planning and effective navigation of unforeseen risks.

To improve raw material management and establish a robust prevention and control framework, there should be a heightened focus on the quality and safety of raw tea materials. Export tea enterprises must exert strict control over key aspects, such as the management of tea plantations, the pesticide registry encompassing procurement and application and the collection of fresh leaves. By initiating stringent controls at the source, the presence of pesticide residues can be mitigated, fostering the standardization of tea garden cultivation.

Institute a traceability mechanism and refine the traceability management system: Export tea enterprises must bolster their information technology infrastructure to enhance the traceability system. This involves closely monitoring the composition of the raw materials used in exported tea and managing the quality and safety of the finished products. Further improvements in the identification process and the establishment of comprehensive production and processing records are essential to engender trust among international customers regarding product quality and safety.

Adopt scientific management practices and actively pursue relevant certifications. To keep pace with the evolving landscape of international trade, export tea companies should enforce stringent internal management protocols and refine their quality management systems. Actively seeking quality management system certifications and leveraging the expertise of system consulting agencies for diverse certification processes will be key to adapting to new trade dynamics.

### 4.2 Developing industries in target markets

From a long-term development perspective, the advantages of China’s tea will continue to be highlighted in different countries and regions to optimize the structure of China’s tea exports. We should further increase the development of tea health products, improve the application of tea technology in tea exports, and improve the added value of tea products. We should pay attention to the development of new-style tea drinks on the basis of the beverage market and enhance the discourse of tea drinks in the tea market.

First, in the context of Russia and CIS countries and other markets, attention should be given to the analysis of their love of black tea. This tea consumption market has stabilized, with the opening of this country and region and the improvement of living standards. To focus on recommending Chinese green tea and special tea and pay attention to the cultivation of consumer habits, the future of Chinese tea exports should continue to increase. Second, in the U.S. market, the U.S. market has diversified tea, green tea, special tea, organic tea and tea health products, and tea products as ideal health drinks and food, with the United States and China’s tea industry continuing to expand exchanges. The situation is positive and continues to rise. The third is the EU market; the EU tea testing standards are relatively strict and, to a certain extent, affect tea exports. Furthermore, in recent years, China has focused on improving quality and efficiency, maintaining strict organic tea garden standards, and national quality inspection departments have strictly prioritized tea export quality; this market will continue to be stable. Fourth, Asia, Africa, Islamic countries and other markets are the traditional advantages of China’s tea export market, and for the countries and regions with strong tea-drinking habits, green tea has become necessary. With the economic development of the countries and regions, China’s tea export situation is good. Fifth, the Japanese market is the traditional main market for China’s tea exports, especially oolong tea, steamed green tea production and export from major countries, and the Japanese market has significant objective demand. Chinese tea enterprises actively respond to changes. Six is the Middle East market, which is also the main market for black tea consumption. Due to local unrest, the impact of China’s tea enterprises expanding the tea market in the region will increase slightly in the future.

### 4.3 Data analysis of Chinese tea industry in recent years

#### 4.3.1 Analysis of tea planting area growth in China

**Fig 5:**
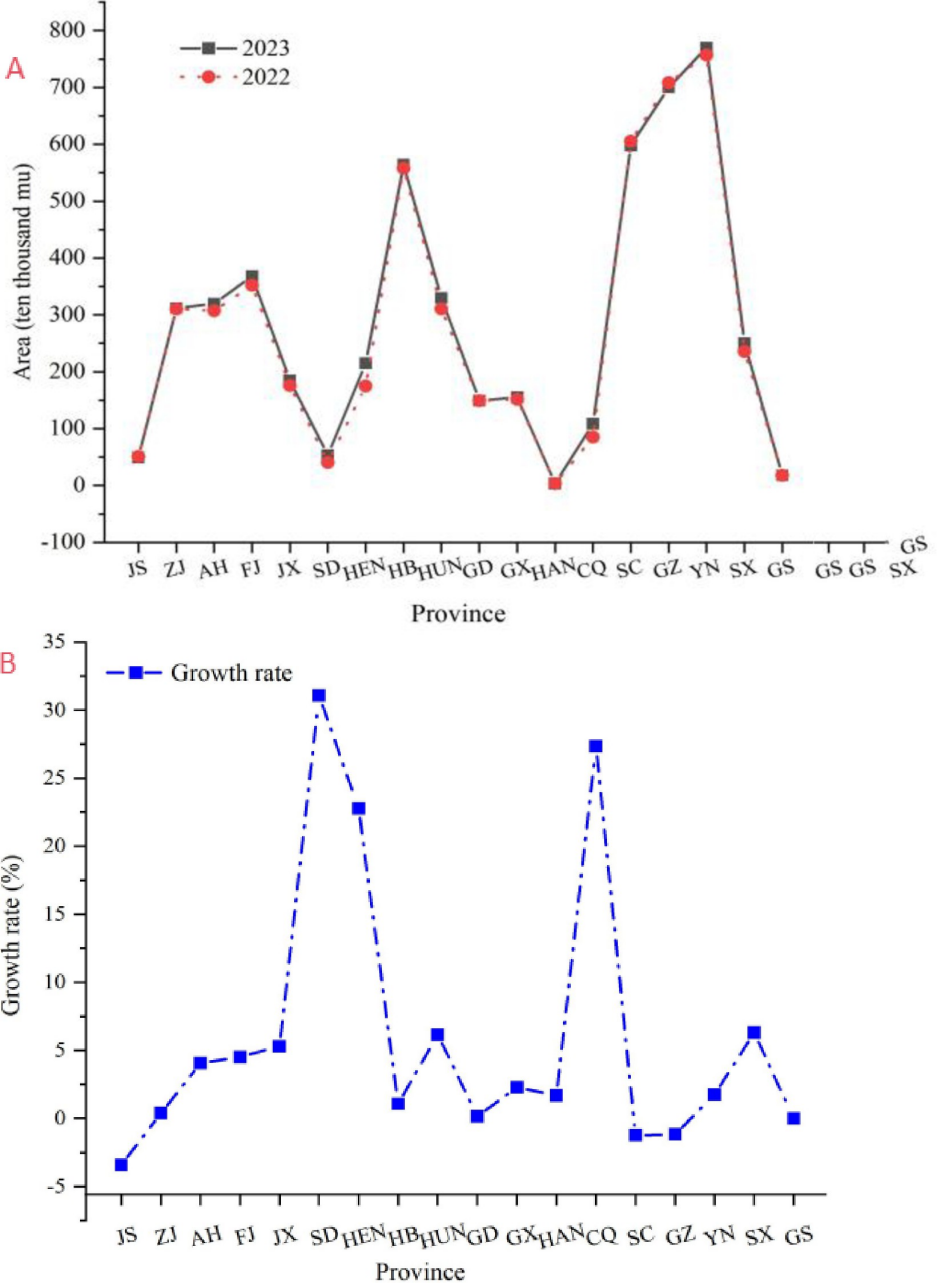
Tea production area and growth rate in China from 2022 to 2023. (a) Comparison of tea area in different provinces. (b) Growth rate analysis.

As shown in Fig 5A and 5B, the analysis presents a comprehensive overview of the growth in tea planting areas across various provinces in China for 2023. The data reveal a pattern of expansion in tea cultivation, with several provinces demonstrating significant growth rates. Notably, Shandong Province exhibited the most substantial increase, with a 31.08% growth rate, followed by Jiangxi Province and Fujian Province, both of which presented growth rates above 4%. Anhui Province also experienced a healthy increase, indicating a positive trend in tea cultivation. Conversely, Jiangsu Province experienced a slight decline, suggesting potential market saturation or a shift in agricultural priorities. The growth in tea planting areas can be attributed to various factors, including government policies, market demand, and advancements in agricultural practices.

The regional variability in tea planting area growth rates highlights a nuanced picture of the development of the Chinese tea industry. While the majority of provinces report an increase, the rates of growth differ significantly. For example, Chongqing experienced a substantial increase of 27.35%, highlighting the potential for further expansion in tea cultivation. The relatively stable growth in Guangdong Province and the slight decline in Guizhou Province suggest a more conservative approach to tea cultivation in these regions. The overall trend indicates a continued commitment to the tea industry, with a few provinces opting for consolidation rather than aggressive expansion. The data underscore the need for tailored strategies that consider regional specificities and market dynamics to ensure sustainable growth and competitiveness in the global tea market.

These insights into regional growth patterns and overall industry trends provide a foundation for policymakers and industry stakeholders to make informed decisions regarding resource allocation, market positioning, and long-term planning in the Chinese tea industry.

#### 4.3.2 Province-level analysis of tea industry value

**Fig 6:**
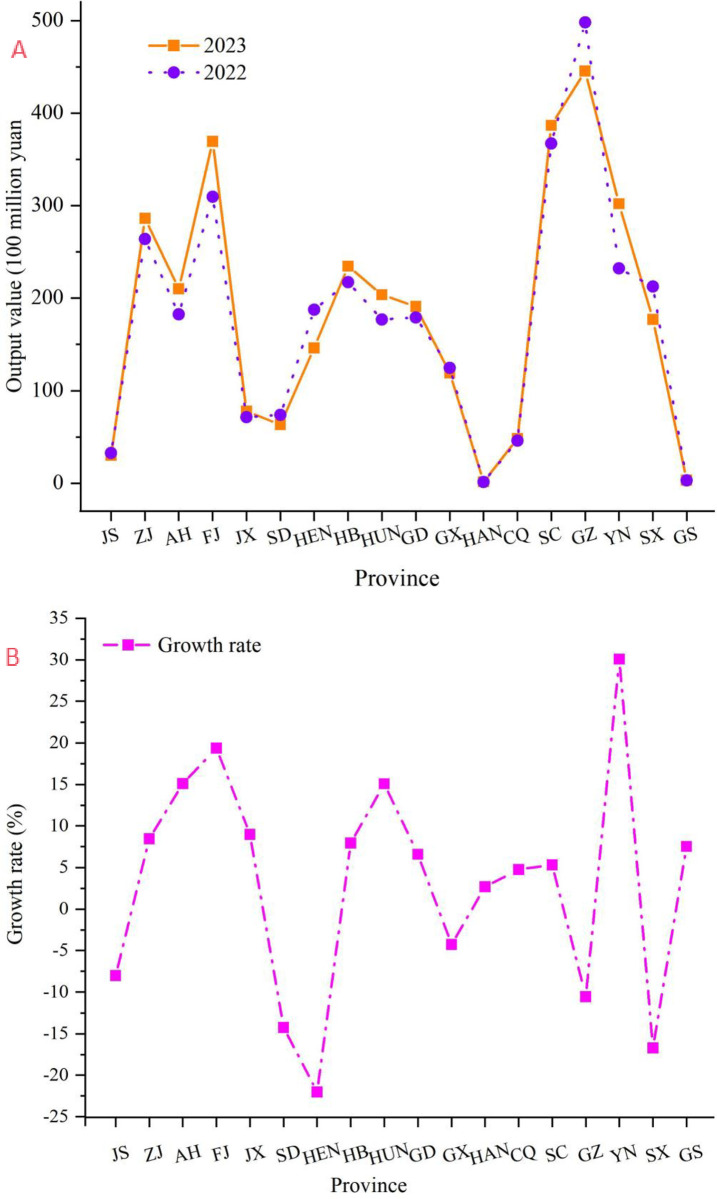
Analysis of tea output value distribution and growth- decrease rate of provinces. (a) Comparison of out-value in various provinces. (b) Growth rate analysis.

The analysis delves into the provincial economic contributions of the tea industry in China, as reflected by the gross value data from 2022–2023, as shown in Fig 6A and 6B. The findings reveal a dichotomy in growth patterns across various provinces. Notably, Fujian Province exhibited the most robust growth, with a nearly 20% increase, underscoring its burgeoning tea industry. Similarly, Yunnan Province experienced a remarkable surge, with a 30.08% growth rate, indicating a significant expansion in tea production and market penetration. Anhui and Hunan Provinces also experienced substantial increases, suggesting a positive trend in the regional tea sector. These growth trajectories can be attributed to a combination of effective agricultural practices, market demand, and provincial policies that favour the tea industry.

Conversely, several provinces, such as Jiangsu and Henan, experienced a decline in tea industry value, with Jiangsu showing an 8.01% decrease and Henan showing a substantial 22.02% reduction. This downwards trend may indicate challenges within these regional tea sectors, such as market saturation or increased competition. Guangxi and Guizhou Provinces also reported decreases, albeit at a lesser magnitude. These declines necessitate a critical examination of the underlying factors and potential policy interventions to revitalize the industry.

The aggregate analysis painted a complex picture of China’s tea industry, highlighting the need for a nuanced approach to regional development strategies. Provinces experiencing growth present opportunities for further investment and expansion, whereas those facing declines require targeted support to address underlying issues. The substantial growth in provinces such as Fujian and Yunnan suggests that with the right combination of agricultural innovation, market orientation, and policy support, the tea industry can continue to thrive. The disparities in provincial growth rates underscore the importance of tailored strategies that consider regional specificities, market dynamics, and the broader economic context. For provinces experiencing decline, there is a need for immediate policy reviews and the implementation of initiatives aimed at enhancing the competitiveness and sustainability of their tea sectors. For those with growth, the challenge lies in sustaining this momentum through continued innovation and market engagement. This study’s findings provide a foundation for policymakers, industry leaders, and researchers to make informed decisions and chart a path forward for the continued prosperity of China’s tea industry.

#### 4.3.3 Integrated analysis of china’s tea industry: Cultivation and economic valuation

The dual analysis of cultivation areas and economic output within China’s tea industry presents a comprehensive view of the sector’s dynamism. On the cultivation front, the data from various provinces indicate a province-specific growth trajectory, with Shandong Province leading in expansion, whereas others, such as Jiangsu and Guizhou, exhibit a retrenchment. This variability in growth rates could be attributed to regional agricultural policies, market forces, and the adaptability of local tea industries. Economically, the industry shows a mixed landscape, with provinces such as Fujian and Yunnan recording significant increases in output value, suggesting a robust and thriving market presence. In contrast, provinces such as Jiangsu and Henan have experienced a decline, which may signal the need for policy reevaluations and strategic interventions to address challenges in the tea value chain.

The synthesis of cultivation and economic data reveals that while some regions are experiencing a renaissance in tea production and market competitiveness, others are experiencing stagnation or decline. This calls for a nuanced and region-specific approach to policy-making and industry development. A combination of factors, including but not limited to agricultural innovation, market demand, and effective governance, clearly plays a crucial role in shaping the trajectory of the tea industry in China. The findings underscore the necessity for strategic and adaptive planning to manage the complexities of this diverse and multifaceted sector.

### 4.4 Analysis of influencing factors restricting the development of tea in China

The high-quality development of China’s tea export industry is a complex and challenging endeavor, which necessitates addressing intense competition from major global tea-producing nations such as India, Sri Lanka, and Kenya, which compete with China in the international market for their lower production costs, stable outputs, and robust brand presence. Additionally, the industry must adhere to increasingly stringent international quality standards and certification requirements, including regulations regarding pesticide residues, heavy metal content, and sanitary safety, which are prerequisites for Chinese tea to gain market access and consumer trust globally. Despite China’s vast tea garden areas and output, which constitute a significant portion of the global total, the international brand influence of Chinese tea remains relatively weak, lacking globally recognized names akin to Lipton, thus limiting its competitive edge in the global market. Consequently, enhancing brand development and implementing effective marketing strategies are essential for increasing the international profile and market share of Chinese tea. Furthermore, optimizing supply chain management is crucial for ensuring the quality and timely supply of tea from cultivation to processing and export. Any inefficiency or mismanagement within the supply chain can lead to a decline in the final product’s quality, thereby affecting market competitiveness.

The lack of technological innovation and R&D investment is also a critical factor constraining the development of China’s tea export industry, as the capabilities in tea processing technology and new product development directly impact the quality and diversity of tea, with innovation deficiencies potentially leading to product homogenization and a lack of market competitiveness. Moreover, international trade policies and barriers, such as tariffs, quotas, and trade protectionism, may restrict the export of Chinese tea, and a lack of domestic policy support and incentive measures may also impact the development of the tea industry. Climate change and environmental factors, including extreme weather events and environmental pollution, may adversely affect tea yield and quality, while the promotion of sustainable agricultural practices is vital for environmental protection, quality enhancement, and meeting consumer demands for sustainable products. Rapid changes in consumer preferences require the Chinese tea export industry to keep pace with market trends and satisfy the diverse needs of consumers. Finally, information asymmetry may result in inadequate international market recognition of Chinese tea, influencing consumer purchasing decisions.

Therefore, the Chinese tea export industry must adopt a range of comprehensive strategies, including strengthening quality control, enhancing brand influence, optimizing supply chain management, increasing investment in technological innovation and R&D, actively responding to international trade policy changes, implementing sustainable agriculture strategies, and adapting flexibly to market changes. Additionally, international cooperation and exchanges should be fostered to improve the global recognition and influence of Chinese tea, thereby overcoming the aforementioned challenges and promoting the industry’s sustained development and enhancement of international competitiveness.

### 4.5 Development strategy

#### 4.5.1 Strengthening the international dissemination and brand building of chinese tea culture

Promoting the international dissemination and brand construction of Chinese tea culture in the context of globalization is particularly important. To increase the international market’s understanding of and interest in Chinese tea, efforts should be made collectively by the government, social organizations, enterprises, and individuals to strengthen the overseas promotion and education of Chinese tea culture. The implementation pathways include utilizing cultural exchange activities, international tea expos, and tea culture festivals to highlight the unique charm of Chinese tea culture; establishing tea culture centers overseas to hold tea art performances and teaching activities; and publishing tea culture content in multiple languages through social media and online platforms to attract young consumers. Moreover, to build Chinese tea brands with international influence, it is necessary to support potential tea enterprises in brand building, encourage product innovation, carry out brand promotion and marketing activities in the international market, and expand brand influence through partnerships and international distribution networks.

#### 4.5.2 Standardization of the tea export trade and market diversification

Enhancing the quality and standardization of tea production is fundamental to the high-quality development of China’s tea export trade. The tea quality standard system should be established and improved, organic tea gardens and sustainable cultivation methods should be promoted, quality management and traceability systems in the production process should be strengthened, and collaboration with international certification organizations to obtain internationally recognized quality certifications. Moreover, market diversification is key to the development of China’s tea export trade. The tea consumption habits of different countries and regions should be studied in depth, targeted market entry strategies should be formulated, free trade agreements and regional economic cooperation mechanisms should be used to reduce trade barriers, and export channels should be expanded.

#### 4.5.3 Enhancing international cooperation, information technology, and talent cultivation

Strengthening international cooperation and exchanges, establishing cooperative relationships with international tea organizations and major tea-producing countries, and jointly promoting the development of the global tea trade are essential. China has participated in the formulation and revision of international tea standards to enhance its say in the international tea trade and has collaborated with international tea research institutions to conduct tea science research and talent cultivation. The application of modern information technology can significantly improve the efficiency and accuracy of the tea export trade. A tea export trade information platform for data sharing and analysis should be established, e-commerce platforms should be used to expand online sales channels, and intelligent tea production and logistics systems should be developed to reduce costs and improve response speed. At the same time, the training of talent in the fields of tea culture and tea trade should be strengthened by offering related courses in colleges and vocational and technical colleges, encouraging students to participate in international exchanges and internship programs, and cooperating with international tea enterprises and institutions for talent development projects, cultivating professionals with an international perspective and professional capabilities.

Through these comprehensive recommendations and implementation pathways, the high-quality development of China’s tea export trade can be effectively promoted, and the status and influence of Chinese tea in the international market can be enhanced.

## 5. Conclusion

This paper posits that while an extensive body of literature exists on the subject of China’s tea export trade, it is predominantly predicated on a constrained theoretical framework for analysis. Concurrently, the extant research lacks a systematic elucidation of the issues at hand and the articulation of pertinent solutions. In this study, a multifaceted approach was adopted, employing complex network analysis, behavioral economics, and diamond-based theoretical constructs to scrutinize the export status of China’s tea industry. In pursuit of fostering the industry’s high-quality development, strategic recommendations are offered. The findings are delineated as follows:

Recently, Chinese tea has been disseminated to 126 countries and regions, with the expansion of China’s tea research institutions and tea cultivation exhibiting a sustained upwards trajectory. Notably, China’s tea garden area constitutes 60% of the global total, and its output exceeds 40% of the world’s production. Nonetheless, the overarching condition of China’s tea exports remains extensive yet not robust, with the tea market value falling short of that of Britain’s Lipton tea.

Synthesizing the developmental trajectory and trends of both domestic and international industries, this paper advances a strategic blueprint for the enhancement of Chinese tea exports. This blueprint encompasses three overarching objectives: management consolidation, risk mitigation, and market orientation. Specifically, it delineates five pivotal strategies: (i) gathering early warning intelligence to bolster product risk management; (ii) instituting a traceability mechanism and refining the traceability management system; (iii) fortifying source management and erecting a robust raw material prevention and control framework; (iv) implementing scientific management practices and actively pursuing relevant certifications; and (v) nurturing domestic tea industry growth by aligning with international market demands for tea.

(3) The analysis of China’s tea industry reveals a complex interplay of regional cultivation growth and economic valuation, highlighting the need for tailored strategies to address the diverse and dynamic provincial trends that shape the sector’s future.

(4) The international promotion and branding of Chinese tea culture necessitate a coordinated approach involving various stakeholders. This strategy should capitalize on global platforms to highlight the uniqueness of Chinese tea and to educate international consumers. Additionally, it is crucial to standardize tea production, diversify export markets, and integrate modern information technology to increase trade efficiency. Cultivating international talent is also key to sustaining industry development, collectively aiming to strengthen the global presence of Chinese tea.
